# In Vivo Measurement of Middle Ear Pressure Changes during Balloon Eustachian Tuboplasty

**DOI:** 10.1155/2018/9519204

**Published:** 2018-09-06

**Authors:** Holger Sudhoff, Philipp Mittmann, Ingo Todt

**Affiliations:** ^1^Department of Otorhinolaryngology, Head and Neck Surgery, Ruhr Universität Bochum, Klinikum Bielefeld, Germany; ^2^Department of Otolaryngology, Head and Neck Surgery, Unfallkrankenhaus Berlin, Berlin, Germany

## Abstract

**Background:**

Balloon Eustachian tuboplasty (BET) is known as a treatment for chronic obstructive Eustachian tube dysfunction (OETD). The precise mechanism of action is not fully understood. Observations in sheep cadavers and human cadavers have shown specific middle ear pressure changes related to BET.

**Methods:**

In this prospective study using a microfibre optical pressure sensor, pressure changes during BET were for the first time monitored transtympanically in five normal human middle ears in vivo.

**Results:**

Middle ear pressure changes during 21 BETs consisted of five stages (insertion, inflation, deflation, withdrawal, and recovery). The highest pressure change occurred in most of the cases during the withdrawal of the balloon catheter. Withdrawal pressure yielded a mean middle ear pressure of 4.76 mmHg (61.89 daPa) with a maximum of 13.88 mmHg (179.55 daPa). Pressure amplitudes capable of causing barotrauma to ear structures were not detected. Internal carotid artery dehiscences were detected as causative of sinusidual pressure changes.

**Conclusion:**

The middle ear pressure changes detected in vivo during BET can be attributed to the balloon inflation. Further human studies with patients affected by OETD are necessary to gain more insight into the mechanism of action of BET to clarify a possible pressure related second mechanism of action of BET.

## 1. Introduction

Obstructive Eustachian tube dysfunction (OETD) is a widely known condition and is clinically well accepted to contribute to the pathogenesis of chronic otitis media and cholesteatoma. Myringotomy and tympanic membrane ventilation tubes are frequently used in treatments for these middle ear pathologies [1] with the disadvantage of not being a targeted therapy and associated with the specific complications like persisting tympanic membrane perforations.

Since the first descriptions of the treatment in 2010 [2], balloon Eustachian tuboplasty (BET) has become a widely used treatment option for OETD with success rates between 36% and 80% [3, 4] and low complication rates [5]. Recently, the superiority of the BET was shown in a prospective study comparing this procedure with conservative treatment (medication) after paracentesis and/or tympanic membrane tubes [6, 7].

So far, questionnaires and tympanogram to discover the treatment's short- and long-term success have been used in studies to analyse the success of the treatment [8, 9].

The underlying mechanism behind the treatment's success has been assumed to be microtears in the cartilaginous part of the Eustachian tube [2] and a decrease in mucosal inflammation and reducing the load of biofilm infections [10].

Since the inflation of the microcatheter in the Eustachian tube has led to middle ear pressure changes, it was assumed that this could cause inner ear barotrauma and subsequent hearing loss [11]. On the other hand, pressure changes have been assumed to be a secondary mechanism responsible for the success of the procedure by loosening middle ear scar formations and mobilizing retractions [12].

Sheep cadaver observations [12] and human cadaver observations [13] have shown that middle ear pressure changes seem not to be associated with the risk of tympanic membrane or round window disruptions [14, 15]. For the exact calculation of risk with regards to this open question, in vivo measurements became necessary. Therefore, the aim of the present study was to observe the occurrence and quantification of middle ear pressure changes related to BET in vivo.

## 2. Materials and Methods

*Pressure Sensor.* To measure intraprocedural pressure changes during BET, a micro-optical pressure sensor was used. The sensor used was a FOP-M260 device with a tip diameter of 0.26 mm from FISO, Quebec, Canada. In short, the tip of the pressure sensor consists of a hollow glass tube sealed on one end by a thin plastic film diaphragm with a reflective surface of evaporated gold. An optical fibre is located at a distance of 50-100 *μ*m from a diaphragm. The optical fibres attached to a light-emitting diode (LED) light source and a photodiode sensor. Light from the LED source reaches the sensor tip of the optical fibre and fans out as it exits the fibre. It is then reflected by the gold-covered flexible diaphragm. The photodiode senses the reflections and pressure changes induce shifts of the diaphragm, which alters the intensity of the reflected light. The sensor is linked to a computer and allows for a temporal resolution of 500 measurements per second. The accuracy of the sensor is down to 0,3 mmHg/ 4 Pa. Mechanically the sensor is highly robust. Evolution software was used to record the pressure changes.

*Patients*. 5 patients (2 men and 3 females, aged between 40 and 67, with a mean age of 56y) undergoing cochlear implantation with regular middle ear pressure estimated based on tympanogram were included. 21 pressure measurements were performed. The study was approved by the ethical board of the Ruhr University Bochum (3226-08). To detect middle ear pressure changes, a myringotomy was performed, in which the pressure sensor advanced through the tympanic membrane and the myringotomy was sealed air tight with fibrin glue.

**Figure 1:**
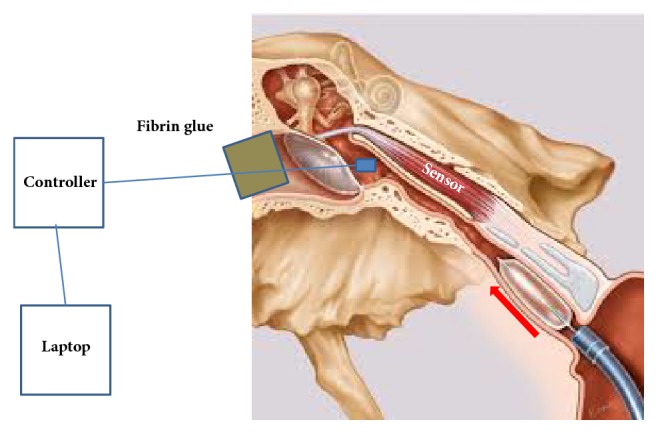
Measurement setting.

*Measurements.* Before transnasal advancement of the balloon and placement within the ET, the sensor was calibrated in the middle ear and the initial value was set to zero. Subsequently, the balloon was inserted. Afterwards the balloon was inflated to 10 bars and held constant for 2 min. The balloon was then deflated and withdrawn from the Eustachian tube, and the pressure changes that occurred were recorded. The procedure was repeated up to 7 times (Figure 1).

## 3. Results

**Figure 2:**
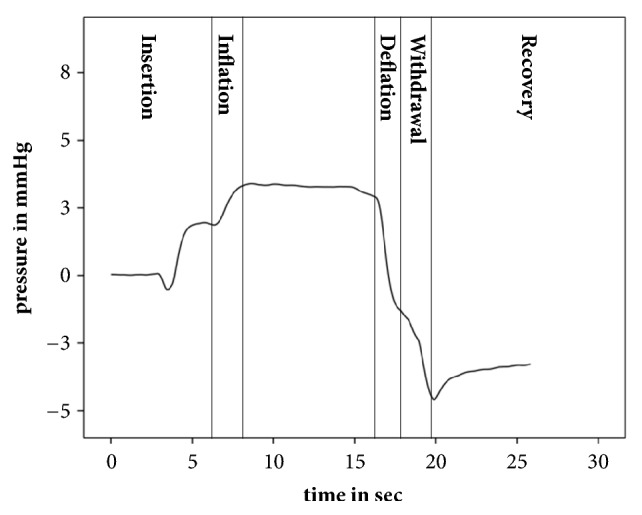
Characteristic stages of middle ear pressure changes during BET.

The pattern of middle ear pressure changes consisted of 5 stages (Figure 2). Insertion of the balloon catheter induced a mean positive pressure change of 1.31 mmHg (17.04daPa) referenced against atmospheric pressure with an initial negative pressure peak. Balloon inflation caused mean positive pressure change of 3.16 mmHg (41.06 daPa). Deflation of the balloon resulted in a mean negative pressure change of 2.14 mmHg (27.79 daPa). Retraction of the catheter induced a negative mean pressure change of 4.76 mmHg (61.89 daPa). Finally, recovery of the middle ear pressure occurred and a mean positive pressure change of 0.82 mmHg (10.62 daPA) were observed.

**Figure 3:**
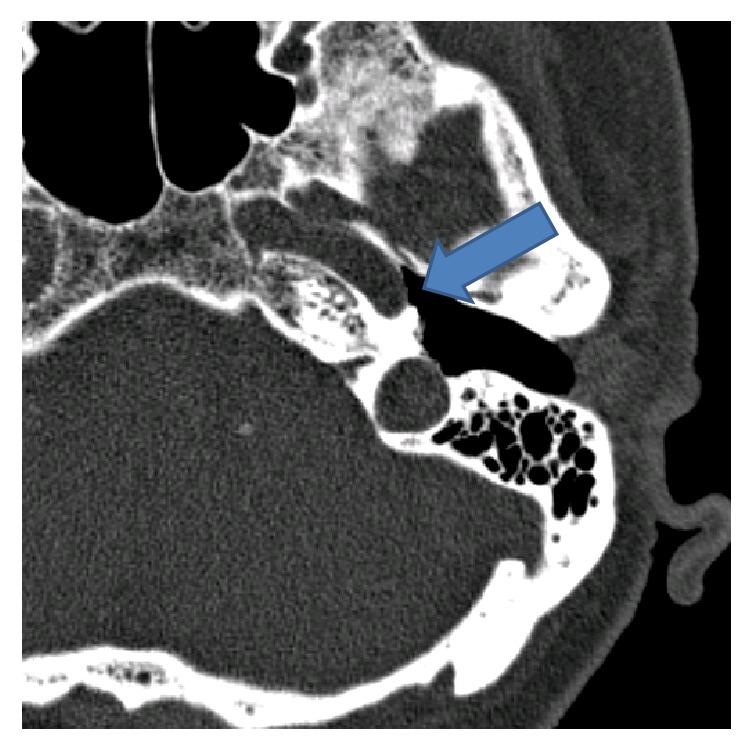
CT scan of a possible bony dehiscence of the internal carotid artery. Arrow indicates dehiscence.

**Figure 4:**
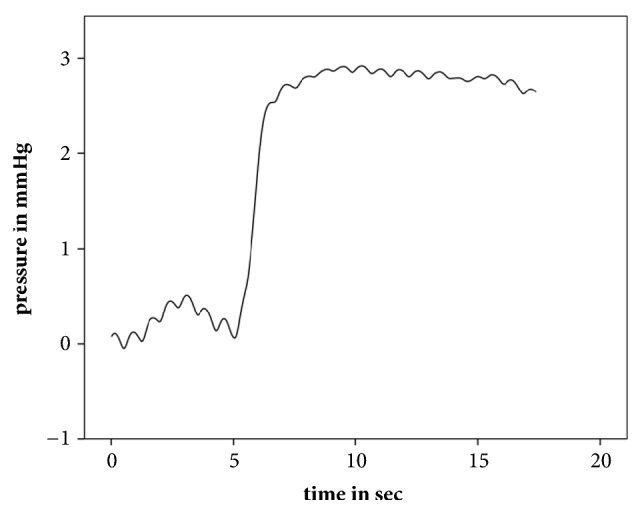
Measured sinusoidal middle ear pressure changes in a case of possibly dehiscent internal carotid artery.

Intraindividual (Table 1) and interindividual variations (Table 2) in pressure changes were observed. Table 1 illustrates the variation in middle ear pressures over four of the insertions in patient 2. In one case, sinusoidal changes of the middle ear pressure were observed (Figure 3). The results of that patient (four measurements) were excluded from the middle ear pressure data analysis (Figure 2). Evaluation of the CT scan in this case identified a possible bony dehiscence of the internal carotid artery (Figure 4) as an explanation for this finding.

## 4. Discussion

BET is a therapy option in chronic obstructive Eustachian tube dysfunction. Recently, a prospective randomized study underlined the success of the procedure [6, 7] and showed the superiority of this treatment compared to conservative (medication) treatment, myringotomy, and middle ear ventilation tubes. Concerning the underlying mechanism, different points can be addressed. Beside a simple widening of the tubal channel and a decrease in mucosal inflammation [10], dilatation-induced microfractures were found [2].

In this human in vivo study, five different stages of pressure changes related to the procedure could be detected (insertion, inflation, deflation, withdrawal, and recovery). This adds additional information to the previous sheep study with its three stages (inflation, withdrawal, and recovery). A possible explanation for this finding could be the difference in the natural tissue reaction to the catheter inflation and deflation by a reactive swelling of the in vivo human tissue, which might differ from the reaction of the tissue of the dead sheep. An initial short negative peak during the insertion stage was observed, which could be related to a positive middle ear pressure release during the initial insertion.

Regarding comparison of the different stages, the highest mean maximum pressure change was observed for the withdrawal of the balloon (4,76 mmHg/61,89daPa). This is in line with the observations made in relation to the sheep model, with the difference being that the mean values in the sheep model were higher than in the human trial (8,52 mmHg vs. 4,76 mmHg). We assume that this finding is related to the smaller volume of the sheep mastoid in contrast with the human trial [16]. The occurrence of pressure is air-chamber-volume dependent, related to the buffer capacity of the middle ear mastoid system. This causative principle might even be the explanation for the interindividual differences in the human trial itself. Additionally, we assume that the intraindividual differences are related to the initial pressure and changes in volume. This even holds true of the interindividual comparison. Similar to the findings of the sheep trial speed-related pressure differences are an additional contributor.

The observed pressure changes even differ from other methods of observing middle ear pressure changes related to ETD in human cadaver models [13]. First, in our in vivo study, an initial insertional pressure peak and a final pressure recovery could be detected. Neither of the peaks were observed in the cadaver study [13]. Additionally, the measured pressure was stable throughout the different stages, which differed from the findings of the cadaver study. The mean pressure change values were comparable to those in the cadaver study, with more variability in the absolute values (e.g., withdrawal, in vivo, SD 77,6 daPa vs. inflation, cadaver, and SD 26,4 daPa). The highest value was 179 daPa for the human study vs. 99 daPa for the cadaver study.

Although static pressure changes related to the procedure are far from occurring at a potentially dangerous level (e.g., Valsalva [14, 15]), it can be assumed that due to variabilities in the affected air volume amount (e.g., small mastoid, mastoid occluding scares, and retractions) and fast or robust handling of the catheter (unblocked withdrawal, high withdrawal speed), complications cannot be fully excluded.

An interesting finding is the observed reflectory pulsatile pressure change in one case measured by the pressure sensor (Figure 4). This finding, in addition to the individual CT finding of a possible dehiscent internal carotid artery (Figure 3), allows us to assume that a dehiscence of the internal carotid arteria is present. Although not representative related to the small sample size, the rate (1 out of 5 patients) of internal carotid artery dehiscences observed in our study should be considered when handling the Eustachian tube. This finding underlines the close relationship between the Eustachian tube and the internal carotid artery [16, 17].

One limitation of this study is the air pressure tight occlusion of the external auditory canal by fibrin glue. Although the middle ear pressure changes created during the procedure remained stable over time, variations in the measured values might be related to pressure leaks that are not fully excludable. Another important point is the limited information about pressure changes in patients affected by an obstructive Eustachian tube dysfunction, as only patients with a normal tubal function were observed in our study.

## 5. Conclusion

The middle ear pressure changes detected in vivo during BET can be attributed to the balloon inflation. Further human studies with patients affected by OETD are necessary to gain more insight into the mechanism of action of BET to clarify a possible pressure related second mechanism of action of BET.

## Figures and Tables

**Table 1:** Mean inter individual pressure changes during the different stages in mmHg. Patient 5 is related to sinusoidual changes (four trials) excluded.

Action/Trial	1.	2.	3.	4.
Insertion	0,25	2	1	1,7

Inflation	0,25	1,5	2,5	2,5

Deflation	0,5	4	3	3,5

Withdrawal	1	4	2	2,5

Recovery	0,5	1	0,5	0,7

**Table 2:** Exemplaric Intraindividual pressure changes during the different stages in mmHg for patient 2 in four trials.

Action/Patient	1.	2.	3.	4.
Insertion	5; 0,2+/-0,12	7; 1,18+/-0,69	1; 3	4; 2,18+/-0,83

*N=X; value and SD in mmHg*				

Inflation	4;0,38+/-0,36	7; 1,54+/-0,87	1; 12,5	4; 1,38+/-0,61

Deflation	3; 0,15+/-0,13	4; 2,75+/-1,55	1; 5,5	4; 0,15 +/- 0,19

Withdrawal	3; 0,07+/-0,06	4; 2,38+/-1,3	1; 13,5	4; 3,1+/- 1,8

Recovery	3; 0	4; 0,68+/-0,24	1; 1	4; 0,78+/-0,56

## Data Availability

The data used to support the findings of this study are available from the corresponding author upon request.
